# The role of sleep in Juvenile idiopathic arthritis patients and their caregivers

**DOI:** 10.1186/1546-0096-12-20

**Published:** 2014-06-03

**Authors:** Karen Tieme Nozoe, Daniel Ninello Polesel, André Campiolo Boin, Laís Fernanda Berro, Gustavo Antônio Moreira, Sergio Tufik, Monica Levy Andersen

**Affiliations:** 1 Department of Psychobiology, Universidade Federal de São Paulo, São Paulo, SP, Brazil

**Keywords:** Juvenile idiopathic arthritis, Sleep, Caregivers

## Abstract

Juvenile idiopathic arthritis is a chronic disease that may lead to various consequences for patients and caregivers, especially in relation to sleep quality. Sleep is an essential process for homeostasis of the organism. In general, caregivers of children with JIA are more susceptible to these sleep disorders and a lower quality of life. This impairment in sleep can potentially affect the health of caregiver. For this reason, it is very important to continue to evaluate the quality of sleep in caregivers and how to support these JIA children’s caregivers more effectively.

## Commentary

Recently an important article was published in the *Pediatric Rheumatology* by Mawani and colleagues, describing the impact of caregiving on parents of children with Juvenile Idiopathic Arthritis (JIA) [1]. We would like to congratulate the authors for their interesting study. The aim of this commentary is to discuss JIA children and their caregivers, particularly regarding the impact of this parental assistance on these parents’ health.

JIA is the most common chronic arthritis disease in children. In the US, 300,000 children younger than 16 years are affected by JIA [2]. This disease may lead to multiple impairments, such as joint deformity, growth impairment and chronic pain. The pain is generally the most distressing of the symptoms. In addition, pain can negatively impact the quality of life of each child leading to physical, social and emotional disabilities. The pathogenesis of pain is multifactorial, and includes genetic, anatomical [3] and psychological influences as well as the contribution of environmental factors [4]. Patients with chronic pain often suffer from sleep disturbances, which affect sleep quality and may increase clinical complaints. Sleep disorders and daytime sleepiness are a result of physiological imbalances that can lead to health problems, such as diminished social, physical and cognitive performances [5].

It is noteworthy that children with JIA may present with poor sleep quality, daytime sleepiness [6], sleep anxiety, night awakenings, parasomnias and sleep disordered breathing [7]. Moreover, patients with JIA have increased risk for sleep apnea, because of changes in facioskeletal features, mainly due to a high chance for posterior rotation of mandible and retrognathia [8]. Sleep is an essential physiologic process for the body homeostasis, especially for the physical development of children and for their immune system. Sleep in childhood corresponds to 40% of the child’s daytime [9], thereby demonstrating the importance of sleep in this stage of life.

This chronic disease and neuro-inflammatory symptoms in children may lead several consequences to their health [10]. There is a bidirectional relationship between pain and disrupted sleep [6], especially regarding sleep impairment, which can exacerbate pain symptoms [11]. However, results from several studies are not conclusive about the mechanism of action of pain in sleep disorder conditions. One possible explanation is that sleep deprivation produces hyperalgesic changes by inducing a decrease of mu- and delta-opioid receptors in the limbic system [12] and reduced affinity of delta- and mu-opioid receptors [13]. Furthermore, sleep deprivation can affect the pharmacological treatment of pain through by inhibiting the analgesic action of painkillers [12,13].

In addition to the sleep-pain cycle, sleep disturbances may lead to other negative health consequences such as impairment and dysregulation of the immune system. Regarding JIA, the patient’s stressful life might be the trigger of an increased inflammatory response [14] that can contribute itself to sleep impairment and the poor sleep may in turn cause an increase in proinflammatory cytokines levels [15]. Thus, sleep deprivation weakens the immune system, changing the activity of immune cells. An important preclinical study showed that sleep deprivation in mice resulted in earlier onset of immune disorders [16], suggesting that sleep impairment could be a risk factor for the onset of any of these disease. We would like to also suggest that a child with undiagnosed chronic pain syndrome that is associated with sleep deprivation should have an appointment with a sleep physician for a sleep evaluation. Thus we must emphasize the importance of sleep for JIA patients. Inside the family circle, the mother often has a fundamental role in the sleep habits of the child.

According with the International Classification of Sleep Disorders, children may have a delayed sleep when their daytime lifestyle is inappropriate or stressful [17]. The mother’s sleep habits may have a strong influence on their children’s sleep as well [18]. In turn, JIA may affect the health of primary caregivers as well, mainly by stress and psychological symptoms [19]. The consequences on the quality of life of primary caregivers still are unclear, but there are some trends such as social, emotional and financial impact in families [20].

Sleep is also affected in caregivers, what can have negative effects on their physical and psychological health [21]. Hence, sleep in caregivers is a relevant aspect that should be considered, especially regarding caregivers of patients suffering from chronic diseases such as JIA. Caregivers often have reduction of total sleep time, increased arousals, insomnia and sleep deprivation [22]. These conditions can potentially affect the health of the caregiver and cause immune [23] and cardiovascular [24] effects, as well as psychological distress [25]. We would like to emphasize that the sleep patterns can vary due to individual situations; parents that take care of children suffering from diseases with a high frequency of requests or need for care by the caregiver may suffer more. Often caregivers are susceptible to burden, burnout syndrome, depression, and a lower quality of life [26] is crucial or the ability to be able to care for the patient or family member will come only with great effort.

The mother-care giver can assist in the sleep hygiene practice and in the development of the physical and psychological health of your child. As the treatment of children with JIA involves a multidisciplinary approach, it should include an evaluation of the sleep quality of the patient and parents, both for the well-being of the patient and their caregivers. Regarding the child, this evaluation should be performed with the integration between clinical history, physical examination and laboratory analysis. Also, the evaluation of the habits and routines of patients over a 24 hour day and night cycle is necessary, focusing on a detailed description of sleep habits on weekdays and weekends (bedtime routine, night time behaviors and naps). Children with JIA suffer from frequent insomnia that may require treatment through cognitive behavioral therapy (CBT). This CBT is recommended by the American Academy of Sleep Medicine [27]. This therapy promotes a reduction of insomnia symptoms and can relieve or reduce pain in children and adults with persistent pain [28].

## Conclusions

Based on the literature cited, we recommend the implementation of professional guidance for children and parents of children with JIA that will improve sleep hygiene and treat sleep problems. These sleep issues are of major importance to caregivers, as well as care providers. We highlight the integrative analysis of the health implications of JIA, regarding both the patient and their caregiver. In this context, the role of experts in integrative and sleep medicine are essential in the guidance and treatment of these families. For this reason, sleep issues should be evaluated for by all care providers of children with JIA and other chronic illnesses and studied more in research. We believe that we may be able to decrease the incidence and severity of these sleep problems and consequently reduce chronic pain in these patients.

## Competing interests

The authors declare that they have no competing interests.
